# A retrospective data analysis of psychiatric cases in Hargeisa, Somaliland between 2019 and 2020

**DOI:** 10.4102/sajpsychiatry.v29i0.1946

**Published:** 2023-02-27

**Authors:** Hassan Abdulrahman, Stephanie Bousleiman, Mustafe Mumin, Ibrahim Caqli, Baraa A. Hijaz, Bizu Gelaye, Gregory Fricchione, Zeina Chemali

**Affiliations:** 1 College of Medicine and Health Science, University of Hargeisa, Hargeisa, Somaliland; 2 Massachusetts General Hospital, Harvard Medical School, Boston, MA, United States; 3 Department of Epidemiology, Harvard T.H. Chan School of Public Health, Boston, MA, United States; 4 The Chester M. Pierce, MD Division of Global Psychiatry, Department of Psychiatry, Massachusetts General Hospital, Boston, MA, United States; 5 Department of Neurology, Massachusetts General Hospital, Boston, MA, United States

**Keywords:** global health, mental health, psychiatry, developing countries, Africa, Somaliland, dual residency programs, unmet need

## Abstract

**Background:**

In Somaliland, an estimated one person in every two households suffers from psychiatric disorders. Despite this, access to mental health care is limited because of shortages in facilities, human resources, funding and stigma.

**Aim:**

To present the proportion of psychiatric disorders encountered in outpatient psychiatry clinics.

**Setting:**

The University if Hargeisa (UoH), Hargesisa, Somaliland.

**Methods:**

De-identified data on patients accessing psychiatric care from doctor trainees in the dual psychiatry–neurology residency program at UoH from January 2019 to June 2020 were included in the analysis. The Institutional Review Board from UoH approved data collection and analysis. The most common psychiatric diagnoses were summarised overall and by sex and age.

**Results:**

A total of 752 patients were included in the analysis. Most were male (54.7%), with an average age of 34.9 years. The most common psychiatric diagnoses were schizophrenia (28.0%), major depressive disorder (MDD) (14.3%) and bipolar disorder type 1 (BD1) (10.5%). When stratified by sex, patients with schizophrenia and BD1 were more likely to be male (73.5% and 53.3%, respectively), and those with MDD were more likely to be female (58.8%). Trauma- and stressor-related disorders accounted for 0.4% of cases, while 0.8% of patients presented with substance use disorders (alcohol and khat), which is an underestimate of the widespread use in Somaliland.

**Conclusion:**

Additional research using structured clinical interviews is needed to determine the epidemiology of psychiatric disorders and promote policies aiming to decrease neuropsychiatric mortality and morbidity.

**Contribution:**

This work presents the first data collection related to neuropsychiatric disorders in Somaliland.

## Introduction

Psychiatric illness constitutes a large portion of the global disease burden and is a leading cause of morbidity worldwide. Over the last several years, there has been an increased focus on quantifying health loss in terms of disability-adjusted life years (DALYs) related to mental health and more generally noncommunicable diseases.^1,2^ In 2016, it was estimated that more than one billion people were affected by mental and addictive disorders globally, which accounted for about 16% of the world’s population. Moreover, these disorders comprise 6.8% of all global burden of disease (GBD) measured by DALYs and account for 18.7% of all years lived with disability (YLD), notably the largest proportion of all larger disease categories. Among psychiatric illnesses, about two-thirds of the DALYs are caused by depressive, anxiety, drug use and alcohol use disorders. Depression was associated with the most DALYs for both sexes, while the second-highest categories in women and men were anxiety disorders and drug and alcohol use disorders, respectively.^3^

It has been well documented that populations that have been affected by conflict-induced humanitarian crises have increased rates of psychiatric illness.^4,5^ The burden of mental illness is also especially high in low-income countries, although studies measuring the exact prevalence and burden of these disorders are lacking.^3^ This is especially true of Somaliland, for which there have been no reliable statistics on the prevalence of mental illness. As health services are delivered by public, private and nongovernmental organisations, there is no country-wide, comprehensive database that provides an accurate view of the region’s health needs or emerging diseases. Moreover, the studies that have been conducted have relied on general population surveys, because of limited diagnostic capabilities and poor data collection and interpretation.^6,7^ The burden of mental illness in Somaliland is estimated to be one of the highest in the world. An observational study conducted by the General Assistance and Volunteers Organization (GAVO), a local nongovernmental organisation, estimated that at least one person in every two households in Somaliland has a mental illness.^7,8^ For Hargeisa specifically, the capital of Somaliland, a 2002 study estimated that 21% of households care for at least one family member with a severe mental health problem.^6,8^

The reasons for such a high mental health burden in Somaliland are multifactorial. Somaliland is a self-proclaimed state in East Africa and is internationally recognised as an autonomous region of Somalia. It has approximately 3.8 million people, over 50% of whom live below the poverty line, with up to 63% of people in rural regions meeting this threshold.^9^ Decade-long trauma from civil wars fought between 1987 and 1996, which involved the widespread displacement of civilians and exposure to high levels of violence, has significantly contributed to the mental health state of the country. There continues to be unearthing of mass graves across the country with seasonal rains.^9,10,11^ Additionally, the widespread use of the amphetamine-based leaf khat has been a large contributor to severe mental illness in Somaliland, contributing to both the onset of psychiatric illness and exacerbation of pre-existing mental illness.^6,7,8,9,10,11,12^ A 2002 survey found that 80% of patients with psychosis excessively used khat before they became ill and that 70% of the patients continued to use this substance even after their diagnosis.^7,12^ Moreover, the high prevalence of female genital mutilation or cutting, estimated to be as high as 98% in Somalia from 2004 to 2015, is suspected of contributing to the mental health burden.^13,14^

Despite the high prevalence of mental illness, access to mental health care is severely curtailed. Most low-income countries invest less than 1% of their health budgets in mental health services.^1^ In Somaliland, there are no functioning mental health structures, and while a national mental health strategy is under development, there is currently no mental health policy in Somaliland.^8^ With the growing demand and need for additional services in the country, the local government has been trying to decentralise all health services, including neuropsychiatric care. Clinics remain poorly staffed because of an insufficient number of trained providers for mental health and suffer from a lack of psychotropic drug supply.^8,15,16^ There are three psychiatrists in Somaliland, all practising in Hargeisa.^17^ Moreover, people with mental illness are highly marginalised and stigmatised.^11,15^ Often, these patients are isolated at home with chains, abused and sometimes imprisoned.^15,18^

Developing Somaliland’s capacity to address the psychiatric and psychosocial problems of the postwar population is crucial and would likely further impact health and economic outcomes.^19,20^ Training in psychiatry was introduced to the medical curriculum in 2007, and since then, there have been increasing efforts to train mental health professionals.^15,21^ In light of the extraordinary need for mental health services, we instituted a dual residency program in neurology and psychiatry with the University of Hargeisa (UoH) in Somaliland to help increase Somaliland’s capacity to carry out evidence-informed brain health care in a sustainable manner and to characterise better health needs and emerging diseases in the region.^17^

In this study, we present an analysis of demographic and descriptive data from patients cared for in outpatient psychiatry clinics at Hargeisa Neurology Hospital over 18 months by three residents of the dual neurology–psychiatry residency program. To date, no studies have reported reliable statistics on the prevalence of mental illness in Somaliland. The objective of our study was to elucidate patients’ access to psychiatric health services in Hargeisa and present the most encountered psychiatric diagnoses. We intended for our preliminary study to offer the first glimpse at psychiatric disorders in Somaliland and what is needed to attend to and promote brain health and neuropsychiatric disease management in this region of the world, enhancing wider community development.

## Research methods and design

### Study setting: Somaliland

Somaliland, a country in the ‘Horn of Africa’ region, is home to an estimated 3.8 million people.^9^ The majority of the population lives in urban areas (52.9%), with the remainder consisting of nomads (33.8%), rural dwellers (11%) and internally displaced persons (2.4%).^22^ Roughly 38% of the population is aged 15 years and below, and 72% are below 30 years of age.^23^ Somaliland is a self-declared state and is internationally considered part of Somalia, a sub-Saharan (SSA) country located in the Horn of Africa.^22^ The capital, Hargeisa, houses 500 000–600 000 people.^22^ In terms of its health infrastructure, the public health care system in Somaliland is still underdeveloped. Twenty-four hospitals, 97 health centres and 162 health posts serve the entirety of the population.^22^

### Study design and study population

A cross-sectional chart review study was conducted to characterise the proportion of psychiatric disease burden in Somaliland. De-identified data from patients cared for in outpatient clinics at Hargeisa Neurology Hospital run by the three residents and their supervising faculty between January 2019 and June 2020 were used in the analysis. Patients were cared for by three resident physicians training in the dual residency program in neurology and psychiatry at the UoH in Somaliland established in partnership with the Chester M. Pierce, MD Division of Global Psychiatry at the Massachusetts General Hospital.^17^ Psychiatric diagnoses were made using the Diagnostic and Statistical Manual of Mental Disorders, Fifth Edition DSM-5 criteria without a structured interview. Patients who had received any type of psychiatric care were eligible for inclusion in our analysis. Patients without a psychiatric diagnosis or with incomplete data (i.e. missing age and sex) were excluded from the main analysis. The Institutional Review Board approved the data collection and analysis in UoH.

### Data analysis

Relevant data were extracted from patient charts, which included patient age, sex and psychiatric diagnosis, to create a database. When applicable, the raw psychiatric diagnoses were classified according to the DSM-5 definition. The proportion of disease burden of psychiatric diagnoses among the patient population was summarised descriptively, and subanalyses were conducted by sex and age groups. A two-tailed *t*-test of proportions was used to determine the differences in the proportion of psychiatric disease burden between men and women.

### Ethical considerations

This article does not contain any studies with animals performed by any of the authors. The Institutional Review Board from the University of Hargeisa reviewed and accepted the work.

## Results

### Patient characteristics

Between January 2019 and June 2020, a total of 752 patients received care at Hargeisa Neurology Hospital psychiatry outpatient clinics and met the inclusion criteria for our study (Table 1). The majority of patients were male (54.7%) with a mean age of 34.9 years. On average, male patients were younger as compared to female patients (mean age ± SD: 33.2 ± 14.6 for male patients; 37.0 ± 18.2 for female patients). Of the 752 patients, 715 had a primary psychiatric diagnosis (395 male patients vs. 320 female patients). The remaining 37 patients had primary neurological diagnoses, including seizure disorders (40.5%), headache disorders (21.6%), musculoskeletal disorders (13.5%) and others (24.3%), and these were not included in the main analyses.

**TABLE 1:** Demographic information.

Participant demographics	Total				Female				Male			
	*n*	%	mean	s.d.	*n*	%	mean	s.d.	*n*	%	mean	s.d.
Number of patients^1^	752	-	-	-	341	45.3	-	-	411	54.7	-	-
**Age**	-	-	34.9	± 16.4	-	-	37.0	± 18.2	-	-	33.2	± 14.6
< 10	14	1.9	-	-	6	1.8	-	-	8	1.9	-	-
10–20	128	17.0	-	-	61	17.9	-	-	67	16.3	-	-
21–30	219	29.1	-	-	87	25.5	-	-	132	32.1	-	-
31–40	168	22.3	-	-	70	20.5	-	-	98	23.8	-	-
41–50	117	15.6	-	-	55	16.1	-	-	62	15.1	-	-
> 50	106	14.1	-	-	62	18.2	-	-	44	10.7	-	-

Notes: 1, Includes patients with psychiatric diagnoses and/or medical diagnoses.

### Trends in psychiatric diagnoses

The most common psychiatric diagnoses were schizophrenia (28.5%), major depressive disorder (MDD) (14.4%) and bipolar disorder type 1 (BD1) (10.5%) (Table 2). Trauma- and stressor-related disorders accounted for 0.4% of cases (Table 3), while 5.2% and 4.5% of patients presented with neurocognitive and neurodevelopmental disorders, respectively. Additionally, 0.8% of patients presented with substance use disorders, including alcohol and khat use disorders.

**TABLE 2:** Most common psychiatric diagnoses among all patients, presented by sex.^1^

Psychiatric diagnosis	All patients (*N* = 715)			By sex						*p*-value
				Female			Male			
	*n*	%	Age, (years, mean)	*n*	%	Age, (years, mean)	*n*	%	Age, (years, mean)	
Schizophrenia	204	28.5	35.6	54	26.5	38.6	150	73.5	34.5	< 0.01
Schizophreniform disorder	18	2.5	29.0	11	61.1	32.2	7	38.9	24.0	n.s.
Schizoaffective disorder	12	1.7	38.3	5	41.7	32.8	7	58.3	42.3	n.s.
Brief psychotic disorder	32	4.5	26.4	17	53.1	25.9	15	46.9	26.9	n.s.
Psychosis (substance induced)	11	1.5	29.2	0	0.0	-	11	100.0	29.2	< 0.05
Psychosis (postpartum)	13	1.8	29.5	13	100	29.5	0	0.0	-	< 0.01
MDD	103	14.4	35.6	61	59.2	35.4	42	40.8	35.9	< 0.05
MDD (with psychotic features)	32	4.5	32.0	8	25.0	29.8	24	75.0	32.7	< 0.05
MDD (with anxious distress and panic)	13	1.8	27.5	8	61.5	29.8	5	38.5	24.0	n.s.
Bipolar 1 disorder	75	10.5	27.1	35	46.7	26.7	40	53.3	27.6	n.s.
GAD	22	3.1	39.1	21	95.5	38.4	1	4.5	55.0	< 0.01
Panic disorder	14	2.0	29.3	7	50.0	31.6	7	50.0	27.0	n.s.
Intellectual disability	24	3.4	14.9	10	41.7	15.0	14	58.3	14.8	n.s.
MNCD without behavioral disturbance^2^	10	1.4	73.5	8	80.0	76.5	2	20.0	61.5	< 0.05
Insomnia disorder	16	2.2	45.3	7	43.8	46.6	9	56.2	44.3	n.s.

Notes: 1, A total of 715 patients with primary psychiatric diagnoses were included in this analysis; 2, Includes MNCD due to AD, vascular dementia, and LBD.

AD, Alzheimer’s disease; GAD, generalized anxiety disorder; LBD, Lewy body dementia; MDD, major depressive disorder; MNCD, major neurocognitive disorder.

**TABLE 3:** Mood, anxiety, obsessive-compulsive disorder and trauma-related disorders.

Psychiatric diagnosis	All patients			By sex					
	*n*	%	Age, (years, mean)	Female			Male		
				*n*	%	Age, (years, mean)	*n*	%	Age, (years, mean)
**Mood Disorders**									
MDD	103	14.4	35.6	61	59.2	35.4	42	40.8	35.9
MDD (with anxious distress and panic)	13	1.8	27.5	8	61.5	29.8	5	38.5	24.0
MDD (with psychotic features)	32	4.5	32	8	25.0	29.8	24	75.0	32.7
MDD (postpartum)	5	0.7	29.2	5	100.0	29.2	0	0.0	-
Bipolar 1 disorder	75	10.5	27.1	35	46.7	26.7	40	53.3	27.6
Bipolar 2 disorder	1	0.1	70.0	1	100.0	70.0	0	0.0	-
**Anxiety Disorders**									
GAD	22	3.1	39.1	21	95.5	38.4	1	4.5	55.0
Social anxiety disorder	7	1.0	20.4	2	28.6	23.5	5	71.4	19.2
Panic disorder	14	1.9	29.3	7	50.0	31.6	7	50.0	27.0
**Obsessive-Compulsive and Related Disorders**									
OCD	5	0.7	34.2	3	60.0	35.0	2	40.0	33.0
**Trauma- and Stressor-Related Disorders**									
Acute stress disorder	1	0.1	43.0	0	0.0	-	1	100.0	43.0
PTSD	2	0.3	39.0	2	100.0	39.0	0	0.0	-

GAD, generalized anxiety disorder; MDD, major depressive disorder; OCD, obsessive compulsive disorder; PTSD, post traumatic stress disorder.

### Psychiatric diagnoses by sex

When stratified by sex, patients with schizophrenia were significantly more likely to be male (73.5% male vs. 26.5% female, *p* < 0.01). Other schizophrenia spectrum and psychotic disorders had varying presentations between sexes (Table 4). Of note, among patients with psychosis, male patients accounted for 100% of patients with substance-induced psychosis. In terms of mood disorders, patients with MDD were more likely to be female (58.8% male vs. 41.2% female, *p* < 0.05) (Table 2). Male patients comprised a greater proportion of patients with MDD with psychotic features (75% male vs. 25% female, *p* < 0.05). Patients with BD1 were more likely to be male (53.3% male vs. 46.7% female), although this difference was not statistically significant. Meanwhile, 6.9% of female patients had a generalised anxiety disorder (GAD) diagnosis, making GAD the fourth leading diagnosis in female patients. Comparatively, only 0.3% of male patients carried this diagnosis (*p* < 0.01).

**TABLE 4:** Schizophrenia spectrum and other psychotic disorders.

Psychiatric diagnosis	All patients			By sex					
	*n*	%	Age, (years, mean)	Female			Male		
				*n*	%	Age, (years, mean)	*n*	%	Age, (years, mean)
Brief psychotic disorder	32	4.5	26.4	17	53.1	25.9	15	46.9	26.9
Delusional disorder	14	2.0	40.1	3	21.4	49.0	11	78.6	37.7
Schizoaffective disorder	12	1.7	38.3	5	41.7	32.8	7	58.3	42.3
Schizophrenia	204	28.5	35.6	54	26.5	38.6	150	73.5	34.5
Schizophreniform disorder	18	2.5	29.0	11	61.1	32.2	7	38.9	24.0
Psychosis	30	4.2	31.9	15	50.0	32.9	15	50.0	31.0
Psychosis (substance induced)	11	1.5	29.2	0	0.0	-	11	100.0	29.2
Psychosis (seizure)	4	0.6	38.5	1	25.0	60.0	3	75.0	31.3
Psychosis (postpartum)	13	1.8	29.5	13	100.0	29.5	0	0.0	-
Psychosis (due to medical condition)	2	0.3	50.0	1	50.0	50.0	1	50.0	50.0

### Psychiatric diagnoses by age

The most common age group among patients with schizophrenia was 21–40 years of age, accounting for 62.5% of the cases of schizophrenia, respectively (Table 5). Most patients with substance-induced psychosis were 21–30 years of age (54.5%). The highest proportion of patients with MDD was among patients aged 21–30 (35.3%). For patients with BD1, the majority were 10–30 years of age (68% of cases). The most common psychiatric diagnoses for patients under 10 years of age were intellectual disability (50%) and autism spectrum disorder (7.1%). Among patients > 50 years old, who accounted for 14.1% of the study sample, the most common diagnosis was major neurocognitive disorder without behavioural disturbance (23.6%).

**TABLE 5:** Most common psychiatric diagnoses among all patients, presented by age category.^1^

Psychiatric diagnosis	All patients		By age					
	%	Age, (years, mean)	< 10	10–21	21–30	31–41	41–50	> 50
Schizophrenia	28.5	35.6	0.0	8.5	29.5	33.0	19.5	9.5
Schizophreniform disorder	2.5	29.0	0.0	33.3	22.2	33.3	11.1	0.0
Schizoaffective disorder	1.7	38.3	0.0	8.3	25.0	16.7	33.3	16.7
Brief psychotic disorder	4.5	26.4	0.0	34 4	43.8	9.4	9.4	3.1
Psychosis (substance induced)	1.5	29.2	0.0	18.2	54.5	9.1	18.2	0.0
Psychosis (postpartum)	1.8	30.4	0.0	0.0	36.4	54.5	9.1	0.0
MDD	14.4	35.4	0.0	15.7	35.3	18.6	14.7	15.7
MDD (with psychotic features)	4.5	33.2	0.0	15.4	30.8	26.9	19.2	7.7
MDD (with anxious distress and panic)	1.8	27.5	0.0	15.4	53.8	15 4	15.4	0.0
Bipolar I disorder	10.5	27.1	0.0	36.0	32.0	18.7	10.7	2.7
GAD	3.1	39.1	0.0	4.5	13.6	45.5	22.7	13.6
Panic disorder	2.0	28.7	0.0	18.2	72.7	0.0	0.0	9.1
Intellectual disability	3.4	14.9	29.2	54.2	12.5	4.2	0.0	0.0
MN CD without behavioral disturbance^2^	1.4	72.7	0.0	0.0	0.0	0.0	0.0	100.0
Insomnia disorder	2.2	45.3	0.0	0.0	25.0	12.5	31.2	31.2

Note: 1, A total of 752 patients with psychiatric diagnoses and/or medical diagnoses were included in the analysis; 2, includes MNCD due to AD, vascular dementia, and LBD.

AD, Alzheimer’s disease; GAD, generalized anxiety disorder; LBD, Lewy body dementia; MDD, major depressive disorder; MNCD, major neurocognitive disorder.

## Discussion

To date, few studies have reported the burden of mental and substance use disorders in SSA, especially in Somaliland. The GBDs, Injuries and Risk Factors Study has been the largest study to present a systematic assessment of psychiatric disease worldwide, although limited data are available for lower-income regions such as SSA.^24,25^ Other survey-based studies have been conducted by a local organisation in Somaliland but have generally had limitations from limited diagnostic capabilities and poor data collection and interpretation.^6,7^ In this study of the proportion of psychiatric disorders in Hargeisa, Somaliland, we have preliminarily begun to characterise the landscape of mental illness in the country, symbolising one of the first undertakings to gain this unique insight in this region of the world.

The greatest proportion of psychiatric disease burden encountered by the residents was schizophrenia, which accounted for 28.5% of patients seen. Moreover, when looking at all psychotic disorders, almost half of all patients are captured, demonstrating the significant burden of this category of mental illness in Hargeisa. A 2004 study conducted in Berbera Mental Hospital, another Somaliland hospital, found that the most prevalent conditions were psychotic disorders, accounting for 32% of patients, although it was unclear if this consisted of inpatient or outpatient cases.^7^ In comparison, the GBD study of 2010 found that schizophrenia was the third leading cause of burden in terms of DALYs, following depressive and anxiety disorders.^25^ Major depressive disorder was the second most common diagnosis, encompassing about one-fifth of patients seen in psychiatry clinics in Hargeisa. This is similar to estimates from Berbera Mental Hospital, which reported 22% prevalent cases of depression.^7^ Moreover, when compared to the GBDs study which ranks depression as having the highest proportion of total disease burden, our study favours schizophrenia.^25^ This is not surprising as our study only included patients accessing psychiatric care. Future large-scale epidemiological surveys are warranted to provide information on the prevalence and distribution of psychiatric disorders. Such studies are essential for developing national mental health strategies for prevention and treatment, particularly for disorders that are more likely to be under-treated and less likely to make it to psychiatric care.

Interestingly, this study found that 0.8% of patients were diagnosed with substance use disorders (alcohol and khat), which is a gross underestimate of the widespread use of khat in Somaliland. Moreover, only 1.5% of patients carried a diagnosis of substance-induced psychosis. The Somaliland Health and Demographic Survey of 2020 described that about 9% of household members in Somaliland reported using khat, but when limited to male household members, this number increased to over 18%.^23^ Moreover, a 2002 study conducted in Hargeisa using home-based interviews found that 31.3% of people surveyed had used khat the week before their interview. The proportion was even higher among those interviewed who also reported psychotic symptoms, which was 46.6%.^26^ The findings in our study do not fully capture the known burden of substance use, particularly khat use, in Somaliland. Indeed, khat abuse–related psychosis may have erroneously been diagnosed as schizophrenia in this sample. In addition, it is possible that although there may be a significant burden of mental illness secondary to khat use, only a limited number of patients present for care. Researchers have noted that a large proportion of people with psychosis in Somaliland are homeless or purposefully kept in hiding by family members, who are afraid to expose them to the public because of many factors, one of which is stigma.^6,26^ This further emphasises the need for increased public awareness and education on mental health, which will increase the likelihood that patients with mental illness will voluntarily present to mental health facilities and families will support social rehabilitation plans for the patients.^6^ These initiatives should be undertaken in parallel with further developing Somaliland’s capacity to care for people with psychiatric conditions.

Another notable finding was regarding trauma- and stressor-related disorders, which accounted for only 0.4% of cases. This is surprising given the severity of the decade-long trauma from wars, displacement of civilians and exposure to high levels of violence among civilians of Somaliland.^10,11^ Studies conducted in similar regions of the world report higher rates of this category of mental illness. A study that analysed common mental disorders in postconflict areas in Africa and the Middle East reported 37.4% post-traumatic stress disorder (PTSD) prevalence in Algeria and 15.8% in Ethiopia.^27^ Other studies in select Somali populations have seen rates as high as 50% of PTSD among the refugee population.^28^ In the study from Berbera Mental Hospital, a smaller percentage was reported (13%), albeit larger than what was encountered in our study.^7^ These discrepancies may be because of differences in health-seeking behaviours among patients in Hargeisa. Although there are few studies on barriers to trauma-related psychiatric care in SSA, a study from South Africa found that the most common barriers to seeking care for trauma-related disorders were structural barriers (i.e. long waiting times at the clinic), shame, stigma, lack of trust in the confidentiality of healthcare workers and seeking out help from alternative sources.^29^

The study had several limitations which are important to be recognised. The analysis relies on quantitative data from chart review and lacks qualitative information regarding barriers to care and other relevant factors to contextualise each patient encounter. It also depends on clinical examination findings and not on a structured clinical interview approach designed to make psychiatric diagnoses systematically. The study was limited to patients coming to outpatient psychiatry clinics at Hargeisa Neurology Hospital. Population-based studies that utilise structured clinical interviews such as the Structured Clinical Interview for DSM-5 (SCID-5) are needed to capture the landscape of mental illness in Somaliland fully. In addition, surveys based on qualitative methods should be undertaken in conjunction with future data collection. Moreover, the availability of age data was limited to the patient’s age at the time of data collection and did not include the patient’s age at the initial presentation. Increased access to this data would allow us to describe trends for when patients first seek mental health care and compare the severity of mental illness based on disease onset. This would also highlight the chronicity of psychiatric disorders and address barriers that may delay presentation. Moreover, patients included in the analysis were those accessing care at specific sites in Hargeisa, limiting the degree to which our findings are generalisable. In future studies, it is essential to better characterise trends in more rural populations and in other areas of the country and gain a more profound sense of the mental health burden of Somaliland as a whole. Lastly, as a retrospective cross-sectional study, our analysis represents a snapshot of the psychiatric disease burden in Hargeisa and would not allow a causal relationship between variables.

Despite the above limitations, our study offered early glimpses at psychiatric disorders in Hargeisa, Somaliland, which can provide a valuable context for developing mental health infrastructure in Somaliland. While Somaliland has made great strides in the postconflict era, current available psychiatric services are inadequate compared with the immense need.^10,11^ Many challenges in establishing such services remain, including the dire need for more trained mental health providers, building capacity and infrastructure and uncovering underestimated disease prevalence in trauma and substance use disorders, including the rampant use of khat.^11^

In conclusion, there is a dire need to improve brain health worldwide and Somaliland is no exception. In that context, our work presents the first data collection related to neuropsychiatric disorders in Somaliland captured by trainees’ clinics. The results contribute substantially to understanding mental health, enlightening clinical progress and policy guidelines in brain health clinics in Somaliland and Africa in general. Future research with mixed qualitative and quantitative methods is urgently needed to fully understand the barriers to adequate access to care, diagnoses, management and follow-up of patients with psychiatric disorders in Somaliland.
